# Emerging role of extracellular vesicles in veterinary practice: novel opportunities and potential challenges

**DOI:** 10.3389/fvets.2024.1335107

**Published:** 2024-01-25

**Authors:** Yindi Xiong, Peng Lou, Chuang Xu, Bo Han, Jingping Liu, Jian Gao

**Affiliations:** ^1^Department of Clinical Veterinary Medicine, College of Veterinary Medicine, China Agricultural University, Beijing, China; ^2^NHC Key Laboratory of Transplant Engineering and Immunology, Frontiers Science Center for Disease-related Molecular Network, West China Hospital, Sichuan University, Chengdu, China

**Keywords:** extracellular vesicles, exosome, biomarker, vaccine, therapy, veterinary medicine

## Abstract

Extracellular vesicles are nanoscale vesicles that transport signals between cells, mediating both physiological and pathological processes. EVs facilitate conserved intercellular communication. By transferring bioactive molecules between cells, EVs coordinate systemic responses, regulating homeostasis, immunity, and disease progression. Given their biological importance and involvement in pathogenesis, EVs show promise as biomarkers for veterinary diagnosis, and candidates for vaccine production, and treatment agents. Additionally, different treatment or engineering methods could be used to boost the capability of extracellular vesicles. Despite the emerging veterinary interest, EV research has been predominantly human-based. Critical knowledge gaps remain regarding isolation protocols, cargo loading mechanisms, *in vivo* biodistribution, and species-specific functions. Standardized methods for veterinary EV characterization and validation are lacking. Regulatory uncertainties impede veterinary clinical translation. Advances in fundamental EV biology and technology are needed to propel the veterinary field forward. This review introduces EVs from a veterinary perspective by introducing the latest studies, highlighting their potential while analyzing challenges to motivate expanded veterinary investigation and translation.

## GRAPHICAL ABSTRACT

Representation of the review structure. This paper discussed EVs potential from tree aspect, biomarker screening, vaccine development, and therapeutic agent.

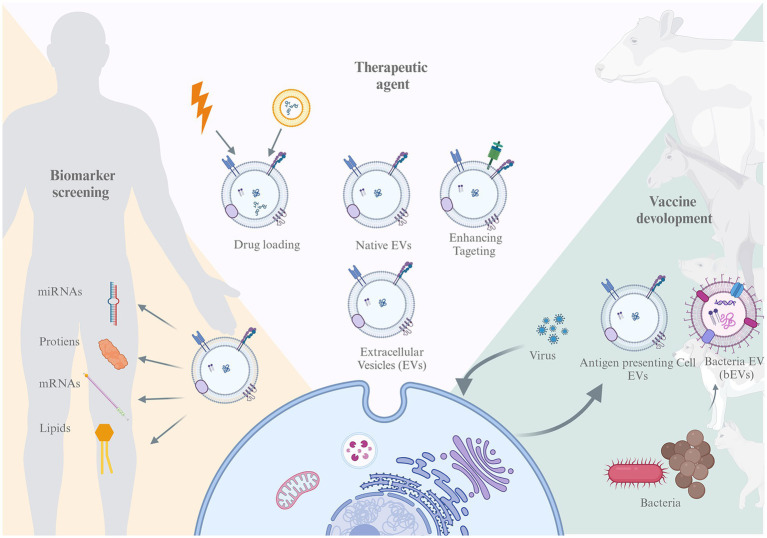

## 1 Introduction

Extracellular vesicles (EVs), characterized by lipid bilayer structures, constitute a diverse group of nanovesicles released by various cell types (1). These vesicles play a crucial role in mediating cell-to-cell communication by transferring a series of cargoes, including proteins, RNAs, DNAs, and lipids, thereby influencing normal physiological processes or pathological conditions (2). Notably, EVs act as biomarkers of multiple diseases, such as cardiovascular disease, cancer, and neurodegenerative disease (3), due to their ability to directly reflect the state of their donor cells or tissues. In addition, an increasing number of studies have shown that EVs from different sources have excellent therapeutic potential in various tissue injuries, including cancer, wound healing, and inflammatory disorders (4, 5). Notably, some products related to EVs are already in different stages of clinical trials. For example, *ExoFlo™*, an EV-based product by Direct Biologic LLC, has decreased the mortality rate associated with respiratory failure or moderate-to-severe acute respiratory distress syndrome (ARDS) due to COVID-19 (6, 7).

Despite the burgeoning interest in EVs within the realms of regenerative medicine and human disease diagnosis, their applicability in veterinary medicine remains relatively understudied. This oversight is attributed, in part, to the unique challenges encountered in veterinary practice, where clinicians operate in the field, relying on clinical signs and diagnostic tools with inherent size limitations. In contrast to human diagnostics, which benefit from readily accessible sampling and analysis settings, veterinary contexts demand rapid responses to prevent economic losses, particularly in the face of swiftly propagating infectious diseases. The imperative for expeditious and accessible diagnostic methods is thus crucial. For the prevention of disease in Veterinary practice, vaccines are one of the important methods. Comparing to traditional vaccine approaches that utilize killed or attenuated agents, EV-based vaccines could guarantee better safety and immunogenicity if utilize properly (8). Furthermore, the prevailing issues of food safety and antibiotic misuse, recognized as key obstacles to the health of both humans and animals, persist, prompting a heightened interest in antibiotic alternatives and targeted medicine within the field of veterinary medicine (9). Therapies based on EVs have shown the potential as strong candidates.

Therefore, the need for noninvasive, easily accessible, and cost-effective disease diagnosis, as well as safe non-hazardous prevention and treatment methods provides EV-based techniques with great opportunities. EVs can be isolated from small amounts of various body fluids, such as blood, urine, and saliva, which can benefit veterinarians when obtaining tissue samples is difficult. The use of native or engineered EVs as treatment agents possesses distinct advantages, including targeted delivery and biocompatibility, which directly address the unique demands of veterinary medicine. Overall, the development of EV technology in veterinary medicine could have great potential and lead to significant advances in the health of different animal species and diseases.

In this review, we focused on discussing the potential use of EVs in veterinary practice by introducing the background of EVs and related research, such as pre-diagnostic techniques, vaccine production, and novel treatments, to provide new insights into animal health. Due to the limitation of scope, only farm animals and pets, but not wildlife and marine species were included in this review.

## 2 Biological properties of EVs

There is sufficient evidence supporting that EVs play crucial roles in cellular and cross-organ communications because their bioactive cargos can regulate recipient cell signaling. In the following section, the biological properties of EVs are briefly introduced, including the biogenesis, isolation and characterization, cellular crosstalk, and cargos of EVs.

### 2.1 Biogenesis of EVs

**Figure 1:**
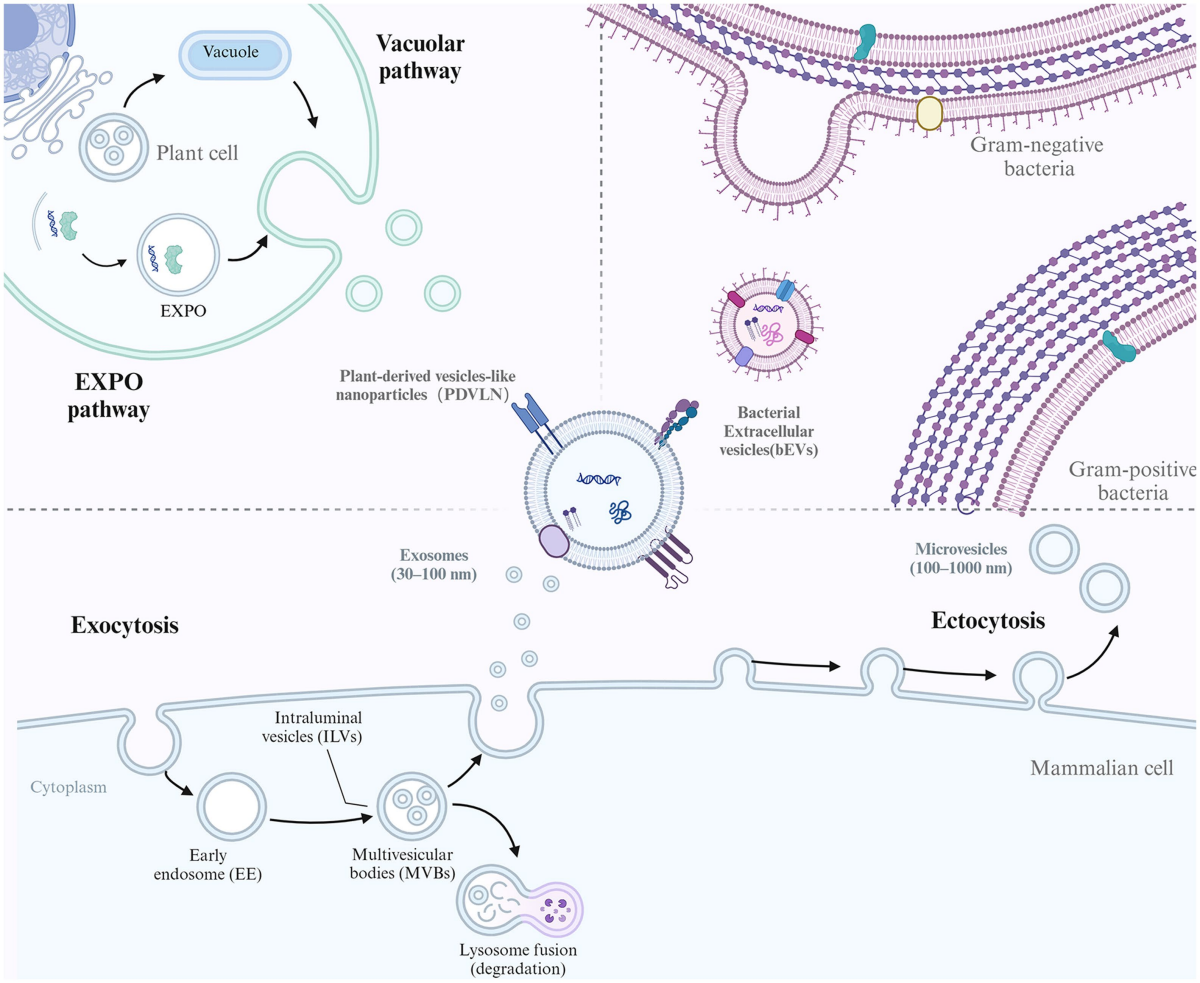
The biogenesis of different EVs. EVs conservatively produced by multiple organisms including mammalian cell, plants cell and Bacteria. For mammalian cell, the biogenesis of exosome and microvesicles (MVs) were illustrated. Exosome biogenesis begins with endocytosis of the plasma membrane to form early endosomes. These endosomes mature into multivesicular bodies (MVBs). The membrane of the MVBs invaginates inward, creating intraluminal vesicles (ILVs). Some MVBs then fuse with the cell membrane, releasing their ILVs as exosomes. In contrast, MVs mainly originate from outward budding of the plasma membrane. For plant cell, EXPO pathway and vacuolar pathway was shown. Bacteria can also secrete EVs as bacterial EVs (bEVs).

EVs comprise a heterogeneous population of membrane vesicles that originate from different cells and are generated via diverse mechanisms. While the exact mechanisms of EVs are not elucidated, some studies suggest that several pathways are involved in forming different EV subtypes (Figure 1). Several subtypes of EVs have been identified based on their sizes and biogenesis, including exosomes (30–200 nm), microvesicles (200–1000 nm), and apoptotic bodies (1–5 μm) (10, 11). Moreover, according to the different sources of EVs, they can also be divided into eukaryotic-derived EVs (e.g., animal tissue/cells and plants) and prokaryotic-derived EVs (e.g., bacteria).

Exosomes are formed by the inward budding of endosomal membranes, resulting in multivesicular bodies (MVBs) (12). MVBs further bud to form multiple intraluminal vesicles (ILVs) and finally fuse with lysosomes for degradation or with the plasma membrane for the release of exosomes into the extracellular space. The sorting of specific biomolecules into exosomes is thought to be mediated by the endosomal sorting complex required for transport (ESCRT) machinery, which recognizes and packages specific cargoes into vesicles (13).

Microvesicles, also known as ectosomes or shedding vesicles, are formed by the outward budding of the plasma membrane (14). This process is thought to be mediated by the cytoskeleton and specific membrane-associated proteins, such as flotillin and tetraspanins. The sorting of specific biomolecules into microvesicles is less well understood than that in exosomes, but it is thought to be mediated by various mechanisms, including direct protein–protein interactions and lipid raft-mediated sorting (15). Apoptotic bodies are formed by the fragmentation of the dying cell’s plasma membrane and the condensation of its cytoplasm and organelles into large vesicles during the process of programmed cell death. The formation of apoptotic bodies occurs in several steps, including cell condensation and nucleus pyknosis, contraction of actin-myosin filaments causing blebbing of the plasma membrane, and large apoptotic bodies pinching off from the protrusions as blebbing continues (16). The sorting of specific biomolecules into apoptotic bodies is thought to be mediated by specific proteins and changes in membrane lipid composition (2).

Bacterial EVs (bEVs) are produced by both gram-negative and gram-positive bacteria and are known as outer membrane vesicles (OMVs) and membrane vesicles (MVs), respectively (17). In gram-negative bacteria, OMVs are released as the outer membrane bulges outwardly, disconnecting from the peptidoglycan layer. Overexpressed and misfolded periplasmic proteins can trigger OMV formation, and the VacJ/Yrb ATP-binding cassette transport system is involved in OMV formation (18). Deletion of the VacJ or Yrb gene results in phospholipid accumulation in the outer leaflet, which causes outward bulging of the OM (19). While the asymmetric expansion enlarged, the OMVs were secreted and enriched in phospholipids incorporated into the outer leaflet of the vesicle membrane (20). Gram-positive bacteria have a thick peptidoglycan cell wall and lack OMVs but were found to release MVs in approximately 2009 (21). The exact process is not clear, although it is hypothesized that MVs could be forced through the cell wall due to internal pressure or enzyme activity weakening the wall (22). For example, *Staphylococcus aureus*-derived MVs contain a peptidoglycan-degrading enzyme that is known to modify gram-positive cell walls (23). Thus, membrane fluidity and cell wall integrity might be the key factors that affect MV release activity.

Plants can also release EVs referred to as plant-derived vesicle-like nanoparticles (PDVLNs) (24). Compared to mammalian and microbial EVs, the biogenesis of PDVLNs is still in its infancy. Several models have been proposed for their secretion. MVB infusion with carrot cells is followed by the release of vesicle-like structures into the wall space (25). Another route of PDVLN release is through the exocyst-positive organelle (EXPO) pathway, a unique plant secretion pathway that was first observed in Arabidopsis (26). In addition, another potential pathway is the vacuolarulolar pathway. As a dynamic organelle of plant cells, vacuoles can release antibacterial and death-inducing proteins outside Arabidopsis cells by fusing with the plasma membrane. While the exact mechanism has not been elucidated, the different mechanisms observed may suggest the heterogeneity of PDVLNs.

### 2.2 EV components and crosstalk with cells

EVs play an important role in cell-to-cell communication through the transfer of their luminal and membrane cargoes. The membrane cargoes that are exposed on the surface of EVs are strictly associated with the membranous structures and interest researchers because they are more accessible to labeling and analytical approaches, while the luminal cargoes are thought to be the bioactive component that comes to therapeutic effects. As a membrane-originated substance, EVs are rich in lipid components such as cholesterol and sphingomyelin, which contribute to the stability of EVs (27). In EV biogenesis, different selection mechanisms contribute to their diverse protein components. Additionally, large amounts of RNA and DNA are observed in EVs (28). MicroRNA (miRNA), which is seen as a crucial part of immunomodulation, is detected broadly in various kinds of EVs. In addition, long non-coding RNA and mitochondrial DNA cargoes of EVs have also gained researchers’ interest. Overall, components in EVs are, by their nature combinatorial and contextually complex.

With an important role in cell-to-cell communication, EVs can interact with the target cell in different ways, including binding to the cell surface, fusing into the plasma membrane, and being taken up by endocytosis. The structure of EVs is similar to that of cells, with extracellular receptors and ligands positioned on the outside and cytoplasmic proteins and nucleic acids on the inside. EVs can directly interact with cognate receptors on the plasma membrane of the cell, which may serve as the targetability of EVs (29). For delivery of the cargo, EVs must fuse into the plasma membrane through endocytosis. Different factors may affect the process, such as the lipid content, environmental pH, and specific receptor-ligand fusion (30, 31). Cells appear to take up EVs by a variety of endocytic pathways, such as clathrin-dependent endocytosis, caveolin-mediated uptake, and micropinocytosis (31). Overall, the interaction mechanism of EVs and cells depends on the protein, glycoprotein, and lipid content on the surface.

### 2.3 Isolation and characterization of EVs

EVs can be isolated from various samples (e.g., blood, tissue, urine, milk) through different methods, including ultracentrifugation (UC), density gradient ultracentrifugation, immunoaffinity, ultrafiltration, and size exclusion chromatography (SEC). UC is one of the most mature methods of EV isolation as the gold standard, which separates EVs based on different sizes and densities using a sequence of centrifuge speeds (32). The exact method differs among researchers and samples, but the purpose could be divided into two parts: low-speed centrifugation for separating cells and debris and high-speed centrifugation for collecting EVs. However, with different speeds of centrifugation, it is difficult to identify EVs among other impurities (protein aggregates, virions, subcellular organelles). In addition, high-speed centrifugation may cause damage to EVs, thus reducing their biological activity (32). Density gradient centrifugation is an improved separation strategy based on centrifugation that uses separation media with different densities, such as iodixanol (33). This process results in a higher purity of EV separation; however, it adds a longer centrifugation time (≥16 h), which limits its clinical application (34).

Ultrafiltration is a separation method based on molecular size, removing impurities through the membrane’s different pore sizes or molecular weight cutoffs (MWCO). For the driving force, electric charge, centrifugation, and pressure can be used. One of the greatest advantages of ultrafiltration is that it can study specific subpopulations of EVs by changing the pore size of the filter to obtain different size scales of EVs. The overall procedure is simple compared to other methods, but if the sample has too many impurities, the clogging of the filtering membrane may cause a low recovery rate.

The immunoaffinity capture method selects EVs through antigen–antibody-specific reactions. As mentioned above, certain proteins and receptors are conserved on the membrane of EVs. These proteins can bind to corresponding antibodies to specifically isolate EVs. Antibodies were fixed on different carriers, such as microfluidic chips and magnetic beads, to capture EVs in fluids. It was reported to have higher separation purity. Immunoaffinity methods suit the needs of researchers who require high purity and specific subpopulations of EVs, while the overall yield is lower.

SEC relies on based on the differential elution profiles of particles of different sizes running through a porous polymer, constituting the stationary phase—also known as gel filtration matrix or resin—and carried through the mobile phase of the SEC column. Compared with other isolation methods, SEC is easier to use in a normal lab setting, and without the process of using high speed centrifugation, it preserved better integrity and bioactivity of EVs. However, the process of SEC is relatively time-consuming compared with fast isolation methods like ultrafiltration, thus limiting the yield and usage (35).

Although the abovementioned traditional methods are most widely used in research, many new separation methods are being developed to address with disadvantages, such as large sample consumption, potential damage to EV membrane integrity, and low purity. Among these new technologies, microfluidics offers a promising platform and demonstrates the virtue of high purity and low sample volumes. Its potential has been demonstrated in some research providing novel diagnostic methods (36).

Current obstacles in EV isolation remain unsolved, requiring a standardized procedure for EV production and overcoming the current high-cost and low-yield challenges. To optimize the yield and purity, multiple methods were combined. There is no single optimal separation method, and the exact method is chosen by downstream application and scientific questions.

According to the minimal information for studies of extracellular vesicles, guidelines require each EV preparation to be tested for morphology, particle size, and marker proteins. To visualize the morphology of EV particles, transmission electron microscopy or scanning electron microscopy is commonly used for analyses. For protein markers, at least three positive protein markers included tetraspanins, ESCRT-related proteins, and at least one negative protein marker (37), are required. However, specific biomarkers of different EV subtypes are not yet clear.

## 3 Extracellular vesicles as biomarkers

Effective monitoring of the health of animals, one of the crucial duties of veterinarians, promotes both welfare and agricultural production efficiency. However, achieving it is difficult due to the complexity of the disease process and limited detection sensitivity. As important mediators of physiological and pathological intracellular communication, EVs carry crucial pathological cargoes from dysfunctional cells and organs and directly reflect the situation of the body. Since EVs can be found in nearly all body fluids (e.g., blood, urine, milk, amniotic fluid), they also provides a noninvasive method of sampling, which suits the need for veterinary diagnoses. Combined with advanced sorting and selection methods, microfluidic chips could provide a promising tool for easy and fast detection (36). Thus, in this section, we will discuss studies identifying EVs as biomarkers for the early detection of diseases that may interest veterinary medicine scientists. Existing studies on biomarker screening for animals are summarized in Table 1.

**Table 1:** Veterinary studies on EVs in biomarkers screening.

Classification	Source	Species	EV subtypes	Status/disease	Type	Biomarker	Refs.
Reproductive	Uterine fluid	*Bos taurus*	Exosome	Endometritis	miRNA	miR-218	(38)
	Serum	*Bos taurus*	EVs	Early embryonic viability	miRNA	miR25, −16b, −3596	(39)
	serum	*Bos taurus*	Exosome	Gestation stage	miRNA	miR499, miR-16a, miR-233 etc.	(40)
	Serum	*Bos taurus*	Exosome	Uterine infection	Protein	CYP, pentraxin, etc.	(41)
	Oviduct and uterine fluids	*Bos taurus*	EVs	Estrous cycle	miRNA	Stage dependent	(42)
	Embryo culture medium	*Bos taurus*	EVs	Embryonic viability	miRNA	miR-1, miR-184, miR-2368-3p etc.	(43)
	Serum	*Bos taurus*	Exosome	Fertility	Proteins	tetratricopeptide repeat protein 41-related, glycodelin, and kelch-like protein 8	(44)
	Cell line culture medium	*Bos taurus*	Exosome	Uterine infection	Characteristic	number or size (Not significant)	(45)
	Seminal plasma	*Gallus gallus domesticus*	EVs	Fertility	Characteristic	HSP90A, portion of small EVs	(46)
	Seminal plasma	*Sus scrofa*	EVs	Semen quality	miRNA	miR-205, miR493-5p, miR378b-3p	(47)
	Seminal plasma	*Sus scrofa*	EVS	Semen quality	miRNA	miR-21-5p	(48)
	Spermatozoa, seminal plasma	*Sus scrofa*	Small EVs	Semen cryotolerance	miRNA	miR-503, miR-130a, miR-9	(49)
	Uterine lavage, serum	*Equus caballus*	EVs	Endometritis	Characteristic	Size	(50)
	Urine	*feline*	EVs	Feline chronic kidney disease, hypertension	Proteins	ANPEP, CES5A	(51)
Metabolic	Serum	*Bos taurus*	Exosomes	Prone to metabolic syndrome	Proteins	α-2 macroglobulin, fibrinogen, and oncoprotein-induced transcript 3	(52)
	Serum	*Bos taurus*	EVs	Diet changes	miRNA	miR-2285bc, miR-2288, miR-3613a	(53)
	Serum	*Bos taurus*	Exosomes	Prone to metabolic dysfunction	Protein	serpin A3-7, coiled-coil domain containing 88A and inhibin/activin β A chain	(54)
Infectious	Serum	*Bos taurus*	EVs	Distinguish tuberculosis and paratuberculosis	Lipoprotein	LpqH	(55)
	Seminal fluid	*Equus caballus*	Exosomes	Persistence *Equus caballus* arteritis virus infection	miRNA	miR-128	(56)
	Milk	*Bos taurus*	EVs	Identify subclinical mastitis	miRNA	miR-223-3p	(57)
	Serum	*Canis lupus familiaris*	EVs	Identify leishmaniasis individuals	Proteins	Myo-inositol and carboxylesterase 5 A	(58)
	Serum	*Canis lupus familiaris*	EVs	Identify leishmaniasis individuals	miRNA	miR-21-5p	(59)
Oncological	Culture medium	*Canis lupus familiaris*	Exosomes	Vincristine resistant	miRNA, proteins	miR-151, miR-8908a-3p, miR-486, CD82	(60)
	Serum	*Canis lupus familiaris*	EVs	Response to CHOP	miRNA	miR-205	(61)
	Serum	*Canis lupus familiaris*	EVs	Glioblastoma	miRNA	miR-15b, miR-343-30	(62)
	Serum	*Canis lupus familiaris*	EVs	doxorubicin-induced cardiotoxicity	miRNA	miR-502	(63)
	Cancer cell line culture medium	*Canis lupus familiaris*	Exosomes	Vincristine resistant	miRNA, proteins	miR-151, miR-8908a-3p, miR-486, CD82	(60)
	Serum	*Canis lupus familiaris*	EVs	Response to CHOP	miRNA	miR-205	(61)
	Serum	*Canis lupus familiaris*	EVs	Glioblastoma	miRNA	miR-15b, miR-343-30	(62)
	Serum	*Canis lupus familiaris*	EVs	doxorubicin-induced cardiotoxicity	miRNA	miR-502	(63)

In this table, we summarized published veterinary studies screening EVs as biomarkers. Studies were summarized according to related classification, source of EVs, species, EV subtypes, disease or status and the exact biomarker. miRNA: micro-RNA.

### 3.1 Reproductive status

Monitoring reproductive status is one of the significant responsibilities of veterinarians. EVs play an important role in both physiological and pathological processes as biomarkers of fertility, embryo quality, placental quality, and early abortion (37).

Fertility is determined by the complex interplay of health and genetic background. According to previous studies, EVs mediate important fetal-maternal crosstalk during gestation and significantly impact maternal-embryo interactions within the reproductive microenvironment. Multiple studies have demonstrated the bidirectional transfer of EVs across the placental barrier between the fetus and maternal circulation (64). In human health, diverse sources of EVs from various maternal sources, including serum, follicular fluid, and plasma, have been isolated and are believed to have the potential to be indicators of female fertility, while miRNAs are the most representative molecules (37). Similarly, in veterinary medicine, particularly in the dairy industry, where reproduction is economically vital, EV-packaged miRNAs have emerged as promising fertility biomarkers (65). A study on cattle showed that specific blood-borne extracellular vesicles carrying miRNAs could be excellent biomarkers of different pregnancy statuses by comparing the different periods of the pregnancy cycle and different embryonic mortality statuses, but the exact miRNA that could serve the role of the biomarker of embryo death and early pregnancy establishment needs further investigation with an expanded sample size (66). Additionally, exosome miRNA profiling of dairy cows was assessed at different periods of pregnancy, including early pregnancy (~60 days) (39). Specific miRNAs for each pregnancy stage were identified, which may be useful for future biomarker inspection. As proposed by Turner et al., specialized analysis of EV contents may provide key insights to improve reproductive outcomes in dairy cows (40). Beyond maternal fluids, EVs have also been isolated from cargoes, implicating dynamic EV-mediated modulation of the female reproductive tract in anticipation of a possible embryo presence (67). Furthermore, *in vitro* studies indicate that bovine embryos themselves secrete EVs during blastulation which may enable noninvasive assessment of embryo viability and detection of early embryonic mortality (42). On the genetic side, heifers bred to have different fertility levels develop extreme diversity in fertility breeding values; however, key animal traits (e.g., body weight, milk production) remain similar. A study reported that through proteome profiling of EVs from heifers with divergent genetic merits, a unique protein expression pattern was observed, suggesting that EV-based prognosis tools could be used to improve cattle fertility (43, 44).

In addition to genetic and health factors, various diseases are also determinants of fertility, contributing to poor reproductive efficiency. For example, a study collected exosomes from the plasma of uterine-diseased cows and tested their capacity to decrease PGF2α production in endometrial epithelial cells, suggesting that the cargo of exosomes could provide a useful basis for the early diagnosis of uterine infection (68). Exosome miRNA-218 was detected in endometritis cows’ uterine fluids and was identified as a potential biomarker for the detection of endometritis (45). In another study, circulating exosomes in dairy cows with or without uterine infection were screened for potential protein biomarkers using high-performance liquid chromatography–tandem mass spectrometry, and71 bovine proteins were found to be unique in infected cows (38). Additionally, a study in equines suggested that the size of EVs from uterine lavage and serum could be a promising biomarker for endometritis in mares (41).

In addition to their emerging roles in female fertility, EVs participate extensively in male reproductive physiology. For breeding animals, the quality of semen is one of the crucial factors for male fertility and economic value. Seminal plasma surrounding sperm is an excellent source for detecting reliable noninvasive biomarkers of sperm quality. EVs isolated from the seminal plasma of chickens exhibited distinct characteristics correlating with male fertility status. Specifically, EVs from fertile roosters were smaller in average size, expressed higher levels of HSP90A, and more readily fused with viable sperm compared to EVs from subfertile males. This implicates seminal EVs as potential indicators of avian male fertility (50). Likewise, miRNA profiling of porcine seminal plasma EVs revealed 15 differentially expressed miRNAs between fertile and subfertile boars. Of these miRNAs, miR-205, miR-493-5p, and miR-378b-3p target genes associated with cellular localization and molecular functions that may impair sperm quality when dysregulated (46). Furthermore, miR-21-5p has been proven to prevent sperm capacitation via vinculin inhibition (47), suggesting that the detection of this miRNA is a negative factor in semen quality control. Beyond the assessment of fresh semen, cryotolerance is also crucial, as cryopreservation enables long-term storage and widespread use of superior genetics via artificial insemination. Long-term semen preservation in mammals remains challenging. Current sperm freezing and thawing methods, even the most effective ones, are suboptimal. Imposing these procedures induces structural, biochemical, and functional changes in sperm. This impairs post-thaw sperm functions such as fertilization capacity (48). In swine, three specific miRNAs encapsulated in seminal plasma EVs, including miR-503, miR-103a, and miR-9, were associated with reduced semen cryotolerance. Detection of these miRNAs may predict cryopreservation outcomes and inform the selective use of higher-quality frozen semen (69).

### 3.2 Metabolic disease

Monitoring for metabolic dysbiosis in livestock herds and controlling the herd-based nutrient balance level is crucial. Most diseases related to metabolism start without clinical signs and are difficult to detect at an early stage. Timely intervention through diet or medication could lead to a better prognosis. The lack of sensitive, noninvasive, and fast-detecting methods is the key challenge for veterinarians, making the development of novel biomarkers important. Metabolic homeostasis emerges from complex, multiorgan crosstalk between adipose tissue, liver, and skeletal muscle. Accumulating evidence supports the role of EVs in metabolic disturbance (49).

Regarding human health, an area of intense research focus involves the emerging metabolic syndromes linked to obesity, nonalcoholic fatty liver disease, diabetes mellitus, and cardiovascular complications. Some studies suggest that the total quantification of EVs in blood may serve as an indicator of metabolic disease progression (70). Additionally, EV cargo such as miRNAs has been explored. One report found that exosomal miR-20b-5b was elevated in patients with type 2 diabetes compared to controls (71). Likewise, another study reported that miR-483-5p encapsulated in serum EVs was associated with obesity and insulin resistance and independently predicted the incidence of type 2 diabetes and cardiovascular events (72). In addition to blood, urinary EVs represent another rich source of biomarkers reflective of metabolic status. For example, Liu et al. identified differential expression of oxidative metabolism-related proteins such as MDH2 in urinary EVs, demonstrating their potential monitoring value (73). Regarding other EV contents, EVs from brown adipose tissue containing 60% proteins were elusive compared to white adipose tissue, including UCP1, Glut1, MIF, and ceruloplasmin, which were suggested to be potential biomarkers of thermogenesis activity (74).

*In vitro*, detected in both human and swine MSCs with metabolic syndrome, suggesting a shared disorder process (75). Circulating exosomes in transition dairy cows were assessed in one study, and specific proteins, including α-2 macroglobulin, fibrinogen, and oncoprotein-induced transcript 3, were identified in exosomes from the serum of high-risk cows that are prone to metabolic syndrome and liver disease (76). In another report on transitional cows, EVs from cows with high-risk metabolic diseases were identified as endoplasmic and catalase proteins, which were absent in EVs from low-risk animals. They were also tested on Madin-Darby bovine kidney cells and found to have a proinflammatory effect (52). In another study, Quan et al. (77) found that the expression of bovine serum EV miRNAs changes according to different diets. Furthermore, exosomes from high and low transitional disease bulls’ serum were identified, and unique proteins, including serpin A3-7, coiled-coil domain-containing 88A, and inhibin/activin β A chain, were detected only in high-risk cows, which indicates good biomarkers for cow health and fertility (53).

While preliminary, these findings suggest the potential for identifying reproducible EV-based biomarkers that distinguish healthy and metabolically compromised animals.

### 3.3 Infectious disease

Current diagnosis methods for infectious diseases rely on detecting evidence of both the pathogen and the host response. On the patient side, pathological changes and the immune reaction toward specific pathogens are tested by serological tests and radiological imaging. On the pathogen side, microbiological culture is the gold standard but can be time-consuming. Additionally, pathogens that cannot be cultured easily escape detection (54). Other methods, including microscopy, require trained personnel with proper sampling methods, and molecular diagnostics, including PCR and immunoassays, which are highly sensitive and specific but require expensive equipment and reagents. Given these limitations, EVs play a crucial role in communication between the host and pathogen, making them great fine biomarkers of infectious diseases.

During bacterial infection, both pathogen and host cells release EVs containing indicators of their status. Bacteria-derived EVs contain bacterial antigens and toxins that can be detected as indicators of infection. Meanwhile, host cell EVs carry an immune response against the pathogen. For example, tuberculosis is a contagious, often fatal, and zoonotic disease caused by *Mycobacterium tuberculosis* (Mtb). The traditional diagnostic methods include sputum culture and molecular diagnostics, such as PCR, for specific genes. These methods are limited due to their high costs and moderate sensitivity (approximately 50%), while microscopic examination is rapid but has low sensitivity (78). Given these shortcomings of existing methods, developing easily detectable biomarkers of active tuberculosis is a global health priority. *In vitro* and *in vivo* studies have shown that Mtb-infected cells release EVs with mycobacterial proteins into cell cultures and body fluids (79). Proteomic analyses have identified candidate biomarkers such as the Cfp2 peptide in patient EVs (80, 81). These findings indicate the potential of mycobacterial proteins carried by EVs isolated from infected cells and body fluids as disease biomarkers, although it has emerged obscurely thus far and should be investigated further (79). Interestingly, one of the candidate molecules, Lpqh, a 19 kDa lipoprotein found in the cell wall of Mtb, relays immunological information to other cells, thereby regulating the host immune response in the pathogen’s favor. This lipoprotein is known to be transported via exosomes secreted from *M. tuberculosis*-infected macrophages. Researchers have reported its application as an efficacious plasma biomarker to distinguish between paratuberculosis and tuberculosis infection in cows. Such EV-based diagnostics could aid tuberculosis clearance in vaccinated dairy herds (82). Another study compared EVs from the milk of healthy and subclinical mastitis cows and discovered that miR-223-3p was upregulated in diseased individuals (55). However, host and bacterial EVs are difficult to differentiate with current processing methods. It is hard to tell whether some authors hypothesize that bacterial molecules identified in EVs during *in vivo* infection may be the result of a mixed population of host and bacterial EVs.

Unlike bacteria, viruses lack cell membranes and cannot directly release EVs. However, viral infections can still modulate host immunity and EV production. In veterinary medicine, there are several reports on EVs from virus-infected animals. For example, EVs from persistent equine arteritis virus (EAV)-infected stallion semen demonstrated downregulation of miR-128, while the expression of CXCL16, a putative target of miR-128, was significantly enhanced (57). However, this pattern is observed in various inflammatory responses, thus making it less credible as a biomarker. In human medicine, it was reported that examination of plasma EVs from HIV-1 patients showed an increased abundance of exosomes and exosome-associated proteins, such as TSG101, in infected patients, compared to uninfected subjects, and this result was also correlated with the CD4/CD8 ratio. The presence of acetylcholinesterase in EVs was further found to correlate with the length of infection, independent of treatment status, disease progression, and patient age, which suggests the use of EVs as biomarkers for HIV infection and progression (56). Overall, research on EVs as viral disease biomarkers is still preliminary, and more work is needed to determine specificity.

Several parasites have been shown to interact with their hosts through intra- and inter-community communication mechanisms, which were identified to be mediated by EVs through various uptake mechanisms (83). For malaria, for example, EVs were identified in infected individuals and were enriched with typical EV markers as well as parasite proteins (73). Two of these proteins, carbonic anhydrase I and S100A8, were verified to be associated with cerebral malaria in both murine and clinical samples, highlighting the importance of EV protein content as a source of protein markers (84). In veterinary medicine, sporadic studies have been conducted to identify biomarkers for parasite disease. A study used proteomic analysis and miRNA sequencing to identify the difference between diseased dogs with Canine leishmaniosis and healthy dogs, stating that several proteins, such as myo-inositol and carboxylesterase 5 A, might be proteins of interest (58, 85). However, most studies only provide description results instead of testified biomarkers.

### 3.4 Oncology

Cancer initiation and progression involve communication between emerging malignant cells and other cell types, often mediated by EVs (59). This makes EVs promising minimally invasive biomarkers for early cancer detection, such as liquid biopsies versus invasive tissue sampling. Although each cancer has a unique progression, all require early diagnosis for optimal patient outcomes. EVs circulating in blood or other biofluids represent an alternative to traditional biopsies for profiling tumors. For example, the protein Del-1 in circulating EVs from breast cancer patient plasma shows potential as a biomarker (86). Additionally, urine EVs have been reported in multiple studies as a source of biomarkers for urological cancers, whether to identify cancer grade (87) or the benign and metabolic state of tumors (88), while their utility for the detection of other cancers is less known. Stool EVs containing human and bacterial RNA could also indicate GI microbiome and tract cancers (89).

In veterinary medicine, oncology studies have mainly focused on companion animals. Early studies isolated and characterized EVs from canine, feline, and human mammary tumor cell lines and analyzed their morphology, protein markers, and size distribution (90). *In vivo*, serum EV levels were significantly higher in canine mast cell tumor patients than in healthy controls (91). Different types of cancer were involved in the research. MiR-15b and miR-343-30 in canine serum-derived EVs could be potential noninvasive biomarkers for canine glioblastoma (62). Additional work profiled miRNA and proteins in EVs from four canine lymphoid tumor lines, finding differential expression of CD82 and certain miRNAs between vincristine-sensitive and resistant cells (92). EVs isolated from canine diffuse B-cell lymphoma patients and dogs also showed increased EV yield compared to healthy animals, with decreases only in dogs with lymphoma (60). Furthermore, analysis of EVs from canine multicentric lymphoma patients receiving CHOP chemotherapy revealed a potential miRNA signature, such as miR-205, correlating with complete/partial response versus no response (93). Additionally, Amelie et al. investigated the correlation between serum EVs and doxorubicin-induced cardiotoxicity, pointing out that miR-502 was upregulated significantly before the third chemotherapeutic dose as a prescient biomarker (61).

## 4 Extracellular vesicles as vaccines

Developing effective measures to halt and prevent the spread of infectious diseases impacting animal husbandry is critical for achieving high standards of quality and welfare in animal production. Vaccine injection is one of the commonly used measures in veterinary practice. While some traditional vaccine approaches utilize killed or attenuated agents, these approaches may have limitations of safety, immunogenicity, and suitability for disease-free populations at risk. EVs present new opportunities for vaccine development given their bioactivity and safety profile. Specifically, EVs derived from pathogens or host cells have potential applications as either direct vaccine candidates or delivery vehicles. Recent work has highlighted several potential benefits of using exosomes as vaccines. First, exosomes can provide stable conformational conditions to maintain the native structure of antigen proteins. Second, their ability to circulate and reach distal organs improves molecular biodistribution compared to conventional vaccines. Third, the expression of adhesion molecules on exosomes enables more efficient antigen presentation to immune cells. Finally, as a natural mechanism for transporting antigens between cells, exosomes may enhance cross-priming of immune responses (63). Exploring the roles of EVs in next-generation animal vaccines could provide improved protection against pressing infectious diseases in livestock, poultry, aquaculture, and other production contexts. Research is needed to evaluate EV vaccine efficacy, scalability, and feasibility compared to existing options. In this section, we will discuss the potential applications of EVs in vaccine development.

### 4.1 Viral disease

Extracellular vesicles act as a double-edged sword because of their ability to carry and deliver molecules to target cells during infection. EVs play a crucial role in the pathogenesis of infection but trigger immune responses to confer protection against pathogens (63). Compared to traditional vaccines produced in cell culture or eggs, EV-based vaccines have many advantages. For example, endogenous viruses in avian-derived cell lines interfere with the structure of the introduced exogenous virus, causing an allergic reaction after vaccination, or glycosylation directly affects the immunogenicity of recombinant protein in eggs, whereas EV-based vaccines can provide a more stable conformational condition for the proteins and provide a more efficient presentation to antigen-presenting cells (63).

Influenza viruses significantly impact both human health and animal husbandry, while existing vaccine options have limitations. Recent research by Anticoli et al. explored an exosome-based influenza vaccine approach in mice. They transfected muscle cells to express influenza nucleoprotein fused to a mutated Nef protein. Purified exosomes from these cells were administered to mice, eliciting robust cytotoxic CD8+ T-cell responses against the nucleoprotein. The cytotoxicity induced appeared sufficient to eliminate both peptide-pulsed and nucleoprotein-expressing target cells. These promising results suggest that this exosome vaccine strategy can stimulate viral antigen-specific immunity (94). Compared to conventional influenza vaccines, the exosome platform provides targeted antigen delivery and potentially improved immunogenicity.

In veterinary studies, preliminary trials have demonstrated that EVs can stimulate cellular and antibody responses for several important animal pathogens. The first animal virus tested was lymphocytic choriomeningitis virus (LCMV). In this study, exosomes from dendritic cells infected with LCMV or loaded with an LCMV peptide did not induce significant CD8 T-cell activation or antiviral protection, suggesting that exosomes have a limited contribution to CTL priming during acute LCMV infection (95). However, studies on porcine reproductive and respiratory syndrome virus (PRRSV) showed some positive results. EVs containing microRNAs designed to target the PRRSV receptors sialoadhesin and CD163 were able to suppress receptor expression and reduce PRRSV viral titers when delivered to *Sus scrofa* cells (96). This demonstrates the potential for exosomes to effectively deliver antiviral microRNA therapeutics and mediate long-lasting protection against different viral strains (96, 97). Additionally, in another study, immunizing pigs with extracellular vesicle-enriched fractions from the serum of PRRSV-recovered animals elicited virus-specific IgG and IFN-γ responses without adverse effects or detectable viral replication, demonstrating the potential of using serum EVs as a safe, immunogenic vaccine strategy against PRRSV. The EV vaccine enabled differentiation between infected and vaccinated animals and showed enhanced immunogenicity when boosted with PRRSV peptides (98). Ongoing studies are exploring EV vaccines for PRRSV, avian influenza, foot and mouth disease, and other major pathogens.

### 4.2 Infectious non-viral disease

As mentioned above, bacteria can secrete bEVs as OMVs and MVs. The bEVs inherit several pathogen-associated molecular patterns (PAMPs), including antigens, from their donor bacteria and, consequently, can induce an immune response against pathogens. This inherent adjuvant-like property of bEVs makes them well-suited for development as vaccines against bacterial infections (99).

BEVs have the potential to be used to develop vaccines for parent bacteria after genetic detoxification (8). Most studies have focused on OMVs from gram-negative bacteria, and some OMV vaccine candidates have been developed at the preclinical stage, including *Vibrio cholerae, Klebsiella pneumoniae,* and *Bordetella pertussis* (100–102). For example, a study used uniformed *K. pneumoniae* OMVs to vaccinate mice subcutaneously and showed significant protection after infection with a lethal dose of carbapenem-resistant *K. pneumoniae*. Likewise, for gram-positive bacteria, a study suggested that extracellular vesicles from gene-edited *S. aureus* could become a vaccine platform and showed benign protection against different strains of *S. aureus*-established murine sepsis (23). For clinical use, During the outbreak of group B strain-specific meningococcal cases in New Zealand, a national vaccination program was performed using an OMV-based vaccine developed by Chiron Vaccines (Siena, Italy), which was shown to have an estimated 80% vaccine effectiveness for children aged 6 months to <5 years (103).

In addition, bEVs are a good source of bacterial antigens. PAMPs enable interactions with antigen-presenting cells (APCs) and allow bEVs to be phagocytosed by APCs. These characteristics make bEVs an adjuvant to traditional vaccines to boost immunity. One study tested OMVs from the ∆*purM Burkholderia pseudomallei* strain admixed with heterologous peptides and demonstrated that, compared to the adjuvants alum and CpG DNA, OMV adjuvants induced more antibody and B-cell responses (104). When co-administered with an influenza vaccine, OMVs with attenuated endotoxicity showed an improved antigen-specific T-cell response and efficacy in mice (105).

EVs are also produced by parasites as a conserved communication strategy and are often referred to as exosome-like vesicles. There is accumulating evidence that EVs are released in parasitic diseases, mediating both parasite–parasite intercommunication and parasite–host interactions (106). Based on this, it is plausible that parasite-derived EVs may serve as vaccine candidates against parasitic diseases. For instance, schistosomiasis caused by *Schistosoma mansoni* results in over 280,000 deaths annually worldwide but lacks a preventive vaccine (107). EVs released by *S. mansoni* adult worms contain miRNAs and proteins involved in host–parasite interactions (108), including several potential vaccine antigens spanning multiple life cycle stages (109). Investigations are also underway on EV-based vaccines utilizing antigen-presenting cells for parasitic diseases. Toxoplasmosis, a globally prevalent zoonosis caused by Toxoplasma gondii, relies primarily on live, attenuated tachyzoites for veterinary immunization (110). A recent study reported that EVs derived from dendritic cells stimulated with *T. gondii* lysate elicited protective mucosal and hormonal immune responses against Toxoplasma infection in an animal model (111). Similarly, a study on *Eimeria tenella*, the parasite of avian coccidiosis, using exosomes from antigen-loaded dendritic cells showed a protective effect on chickens (112), suggesting the potential of EVs as a protective vaccine production.

## 5 EVs as therapeutic agents

**Figure 2:**
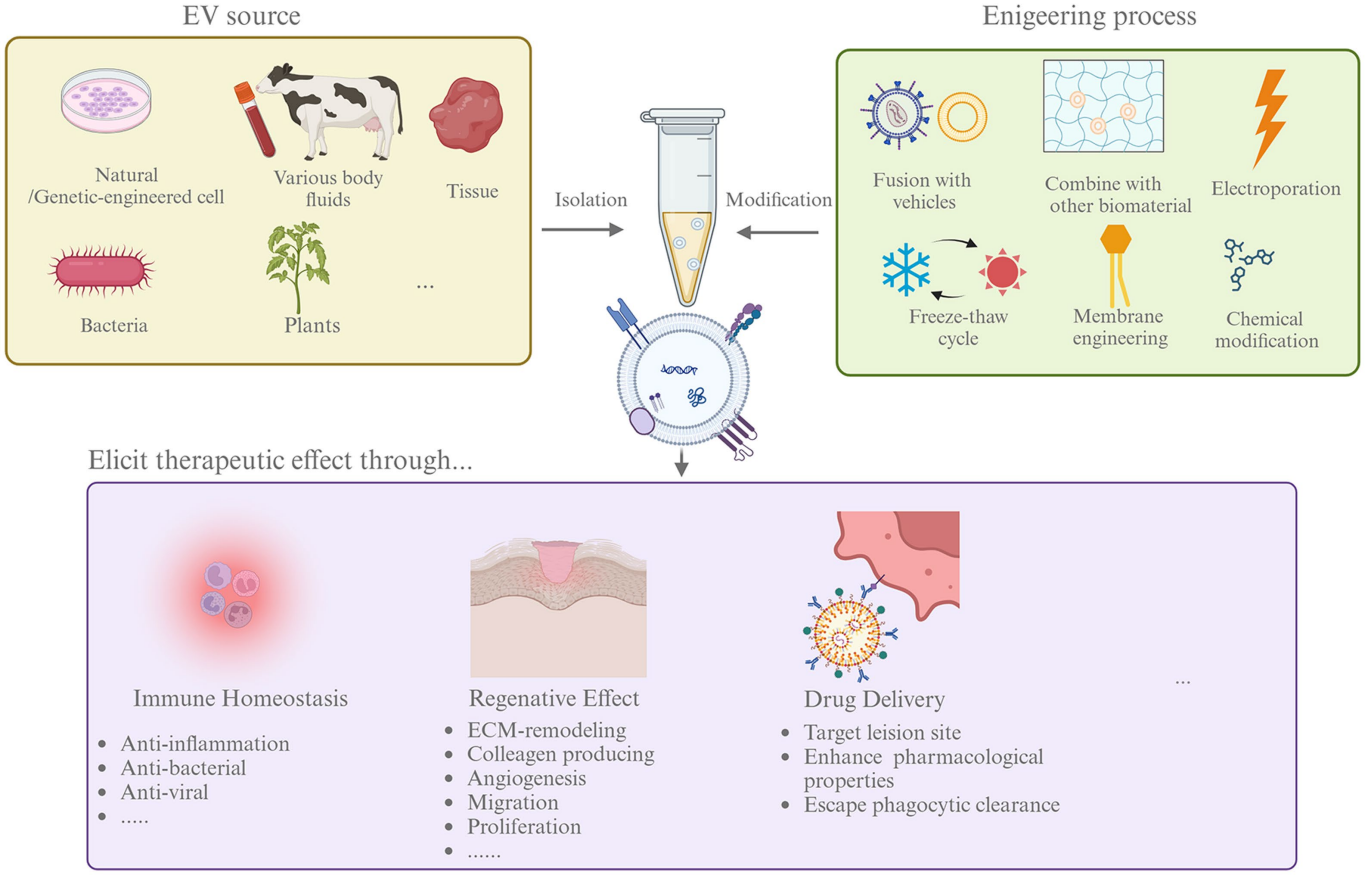
The process of EVs as treatment agents. Therapeutic EVs can be isolated from multiple sources including cultured cells, body fluids and tissue, bacteria, and plants. To enhance the therapeutic effects, EVs can go through different modification by engineering, like fusion with liposome and electroporation. Then, native, or engineered EVs was used in therapies. EVs could affect disease through modifying immune homeostasis, promoting tissue regeneration, and deliver therapeutic agents effectively.

The application of harnessing EVs in the treatment of diseases in multiple fields, including cancer, cardiovascular disorders, and regenerative medicine, is now being recognized (3). However, the studies in veterinary medicine on therapeutic effects are still preliminary, and we summarized existing studies in Table 2. The overall research on EVs in therapy could be divided into research on native and engineered EVs, which will be discussed below (Figure 2).

**Table 2:** Veterinary studies on EVs in therapeutic effect.

Species	EVs origin	EV subtype	Disease/model	Therapeutic effects	Refs.
*Canis lupus familiaris*	MSC	Exosomes	Cutaneous injury	Accelerate wound healing	(113)
*Mus musculus*	Adipose tissue-derived MSCs	EVs	DSS-induced colitis	Relief colitis symptoms	(114)
*Equus caballus*	5-azacytidine and resveratrol pre-treated MSC	MVs	Ligament injury	Increase lesion filling and improve angiogenesis and elasticity in injury tissue	(97)
*Canis lupus familiaris*	*Canis lupus familiaris* M1-polarized macrophages	EVs	melanoma and osteosarcoma tumor cell line	Induce apoptosis and increase the level of pro-inflammatory cytokines	(115)
*Canis lupus familiaris*	Human PRP	Exosome	Dexamethasone treated tenocyte	Reduce cellular apoptosis	(116)
*Equus caballus*	MSC	EVs	IL-1 and TNF-α treated chondrocyte	Decrease inflammation	(117)
*Canis lupus familiaris*	MSC	EVs	renal ischemia–reperfusion injury	Attenuate renal dysfunction, inflammation, and apoptosis	(118)

In this table, we summarized published veterinary studies using EVs as therapeutic agents according to species, EVs origin, EV subtype, disease/model, and the therapeutic effects when applied. MSC, Mesenchymal stem cell.

### 5.1 Native EVs

Native EVs from multiple sources have shown therapeutic effects. Most veterinary medicine research has focused on using native EVs for treatment (119). We will discuss the different sources of native EVs in the following section (Table 2).

#### 5.1.1 Stem cell-derived EVs

Stem cells are attractive to human and veterinary medicine for their propriety of regenerating functional tissue. However, in stem cell therapy, immunogenicity, heterogeneity, and tumorigenicity poses three obstacles, and EVs derived from *in vivo* culturing stem cell could be one of the solutions. In particular, mesenchymal stem cell-derived exosomes (MSC-exosomes) not only have immunomodulatory and tissue regenerative capacity but also exhibit immune privilege that is appropriate for therapeutic approaches (120). In addition, MSC-exosomes can avoid pulmonary embolisms associated with MSC-based cell therapy. Additionally, MSC-EVs cannot proliferate like cells, which will lead to fewer worries about cancer development. It has been shown that MSC-EVs have a similar therapeutic effect to MSCs themselves. For example, MSC-EVs promote M2 macrophages via PGE2 activation in a manner similar to MSCs (121). In a study using dogs as a cutaneous injury model, MSC-MVs had superior capability in wound healing than their originator cells (113). Another strong therapeutic candidate is adipose tissue-derived mesenchymal stem/stromal cell-derived EVs, which have been tested in a murine model of DSS-induced colitis in a murine model (114) and canine renal ischemia–reperfusion injury (118). An *in vivo* study tested the anti-inflammatory effect of equine MSC-EVs on chondrocytes, suggesting their therapeutic potential (117). To enhance the therapeutic effect, EVs from preconditioned cells were also investigated. A case report on treating a suspensory ligament injury in a horse using MVs derived from mesenchymal stem cells pretreated with 5-azacytidine and resveratrol. The anti-apoptotic and proliferation-promoting effects of MVs were tested *in vivo* and *in vitro* (97).

#### 5.1.2 Blood-derived EVs

Circulating EVs from neonatal individuals have also demonstrated therapeutic functions. In 2019, Yoshida et al. reported that EVs from young mice could delay aging and extend the lifespan in aged mice (122). Thus, the effects of circulating EVs on disease are of research interest. For example, one study found that intramyocardial delivery of plasma exosomes isolated from neonatal mice helped repair adult heart’ structure and function after acute myocardial infarction by promoting angiogenesis (123).

Platelet-rich plasma (PRP) has been used in regenerative medicine for more than 30 years, and numerous encouraging outcomes have been obtained. PRP has shown its capability to repair cardiac disease, skin wounds, musculoskeletal disease, and hair growth (124, 125). However, some limitations still need to be considered. For example, PRP-derived biomolecules may be damaged by lytic enzymes from the extracellular environment once released from platelets. PRP-EVs are expected to be more efficient and safer clinical candidates in therapies, especially in regenerative medicine. Most studies on PRP-EVs are on the hemostasis and angiogenesis capabilities of regenerative medicine, while the effect of PRP-EVs on inflammation is biased (126). In veterinary medicine, a pioneering study reported that commercially purified PRP exosomes (human-sourced) can increase cell proliferation and deposition and reduce cellular apoptosis caused by dexamethasone *in vitro* in canine tenocytes (116).

#### 5.1.3 Immune cell-derived EVs

Inflammation is the body’s defense mechanism and helps clear infections; when it becomes out of control, it can lead to harmful outcomes, such as cytokine storms causing organ damage. Immune cells such as lymphocytes, macrophages, and mast cells are involved in the immune response. Crosstalk between these cells is crucial in immune homeostasis, and the EVs from those cells are part of this function. Utilizing EVs from immune cells for therapeutic purposes for the disease may be promising.

While current anti-inflammatory drugs target a single pathway, immune cell-derived EVs provide multitarget therapeutics to modulate immune status. Wang et al. reported that p extracellular vesicles derived from peritoneal M2 macrophages (M2-EVs) exhibited superior anti-inflammatory potential by effectively reducing excessive cytokine release, thereby attenuating oxidative stress and multiple organ damage in endotoxin-induced cytokine storms (127).

In cancer treatment, the crosstalk between the tumor microenvironment and immune cells is crucial. For macrophages, M2-polarized pro-tumoral macrophages (TAMs) express the interleukin-4 receptor (IL4R) at higher levels thanM1-polarized, anti-tumoral macrophages. Thus, using M1-polarized macrophage-derived EVs could be a strategy to modify tumor microenvironments. One single study of the proapoptotic effect of extracellular vesicles derived from canine M1-polarized macrophages on canine melanoma and osteosarcoma cell lines suggested that it could be an effective anticancer therapeutic approach (115). In addition, the polarization of macrophages is reversible, and transforming TAMs to a tumor-suppressive M1 phenotype is an important approach for tumor therapy (128). An M1-exosome was transfected with NF-κB p50 siRNA and miR-511-3p to enhance M1 polarization and surface-modified with IL4RPep-1, an IL4R-binding peptide, to target the IL4 receptor of TAMs (named IL4R-Exo (si/mi)). IL-4R-Exos suppress the growth of tumors by reprogramming TAMs into M1-like macrophages and increasing antitumor immunity, indicating a novel cancer immunotherapy (129).

Dendritic cells (DCs), as crucial antigen-presenting cells, play a vital role in disease pathogenesis. DC-derived EVs were found to harbor functional peptide–MHC complexes, T-cell costimulatory molecules, and other molecules essential for antigen presentation, immune cell regulation, and simulation of immune responses (130). For immature DCs, EVs are an attractive candidate for immunosuppressant therapies for autoimmune and inflammatory disorders due to their immune suppressive capabilities. It has been reported in different disease models in mouse collagen-induced arthritis models (131), colitis (132), and IBD (133). For mature DCs, in their nature as antigen-presenting cells, In viral infection, DC-derived EVs carry viral antigens and particles with detrimental effects on the immune system, transmitting pathogen particles and aiding viruses in escaping the immune system. However, evidence for their protective effect is lacking. However, DC-derived EVs may help lead to a protective immune response against bacterial and parasitic infection, and the effect seems to be pathogen-specific. For example, DCs infected with *Chlamydia psittaci* display enhanced exosome release. The exosomes facilitated the activation of NK cells and upregulated IFN-γ secretion, reducing *C. psittaci* growth and increasing apoptosis in infected cells, thus clearing further infection (134).

#### 5.1.4 Tissue-derived EVs

EVs from *in vitro* cultured cells, such as MSCs, showed certain tissue repair potency. However, the efficacy of current EV therapies is unsatisfactory for multiple reasons, such as the importance of large-scale production and the time consumption involved. Moreover, long-term *in vitro* culturing of cells may affect the phenotypes of the donor cell, thus impairing EV biofunction. Thus, tissue-sourced EVs are a better alternative for treatment (135). Lou et al. reported that neonatal tissue-derived EV therapy is a potent strategy for precision tissue repair in multiple injury models, including skin wounds, acute kidney injuries, and partial nephrectomy (5).

#### 5.1.5 Vesicle-like nanoparticles and bacterial EVs

Vesicle-like nanoparticles derived from edible plants, such as daily vegetables, have demonstrated crosstalk with mammalian cells. Moreover, the oral administration of plant-derived vesicles has been proven safe. Multiple studies have shown that plant-derived extracellular vesicle-like nanoparticles (PDVLNs) can exert therapeutic effects on human diseases. For instance, one study found that PDVLNs derived from grapefruit can be taken up by intestinal macrophages, inducing the expression of the antioxidant gene HO-1 while suppressing the production of proinflammatory cytokines. This ameliorated dextran sulfate sodium-induced colitis in a mouse model (136). While no veterinary research on PDVLNs has been conducted yet, traditional herbal medicines may offer another rich source of bioactive PDVLNs. The clinical benefits of traditional Chinese medicines are often attributed to their multicomponent, multitarget mechanisms of action. Thus, studying PDVLNs may elucidate the therapeutic mechanisms of these traditional remedies (137). For example, one study showed that ginseng-derived nanoparticles can alter macrophage polarization to inhibit melanoma growth (138).

### 5.2 Engineered EVs

The therapeutic potential of various native EVs has been well established. To further optimize their performance and harness their natural abilities, researchers have worked to equip EVs with new targeting and therapeutic moieties while preserving vesicle integrity. For instance, some studies have functionalized EVs with targeting ligands to enhance their specificity for desired cell types or tissues. Other efforts have focused on loading EVs with therapeutic nucleic acids, proteins, or drugs to augment their treatment capabilities.

#### 5.2.1 Enhancing targeting ability

While EVs demonstrate an inherent ability to preferentially interact with target cells *in vitro*, this targeting efficiency is often diminished *in vivo.* For example, Smyth et al. reported that unmodified tumor-derived exosomes exhibit no improvement over liposomes in tumor accumulation or circulation time (139). Moreover, multiple studies note a predominant accumulation of systemically administered EVs in the liver and spleen (140). To enhance EV targeting, researchers have explored modifying vesicles with specific targeting ligands. Strategies to engineer the EV surface fall into two categories: pre-isolation techniques that manipulate the donor cell, including genetic, metabolic, and membrane engineering approaches, and post-isolation techniques that directly modify purified EVs using physical or chemical methods (141).

One of the advantages of EVs is that the editing of parent cells could directly affect EV production and properties. Genetic engineering strategies for surface functionalization of EVs are based on transgene expression of proteins or chimeric proteins containing protein domains that are known to be enriched in EVs. For instance, Mentkowski et al. engineered cardio-sphere-derived cells to express a chimeric protein consisting of Lamp2b fused to a cardiomyocyte-specific peptide (142). The resulting EVs displayed enhanced targeting to cardiomyocytes and improved therapeutic effects in a heart injury model (142). Beyond modifying EV proteins, the glycocalyx is another exploitable target for engineering. Zheng et al. developed a method to co-express glycosylation enzymes and glycoprotein domains in parent cells (143). Specifically, they inserted a glycosylation domain into the extracellular loop of CD63 along with the enzymes FUT7 or FUT9. This approach enabled the display of the glycans sialyl Lewis X or Lewis X on engineered EVs, conferring high specificity for activated endothelial cells or dendritic cells, respectively (143).

Surface engineering of EVs is also possible after isolation, although such post-isolation techniques require additional steps and are more time-consuming than genetic engineering approaches. Post-isolation engineering strategies fall into two main categories: physical interaction methods and chemical modification methods. For physical modification, lipophilic components such as liposomes are often used to fuse with the EV membrane, employing techniques such as freeze–thaw cycling, electroporation, or altering culture pH and salt concentrations. A novel technique-engineered surface of EVs by adsorption. For the chemical approach, EVs were modified through the covalent linkage of molecules to their surface (141). While physical and chemical engineering offer certain advantages, genetic modification of parent cells remains the predominant strategy for EV engineering given its superior controllability and the stability of modifications.

#### 5.2.2 Delivery of therapeutic reagents

Currently, the two mainstream drug delivery systems are liposomes and polymeric nanoparticles (144). Liposomes comprise phospholipid vesicles that can encapsulate both hydrophilic and hydrophobic molecules. Polymeric nanoparticles are synthesized from biocompatible and degradable materials such as natural collagen or synthetic polyacrylates, which facilitating drug transport and extend the drug’s half-life (145). However, the use of liposomes has been reported for rapid clearance via the reticuloendothelial system, and their fast accumulation in the liver and spleen limits the effective concentration of drugs. On the other hand, although polymeric nanoparticles as a delivery platform may have better stability than liposomes, concerns about their safety for long-term use remains (146). Compared to these existing platforms, EVs demonstrate enhanced stability in biological systems and can distribute over short and long distances, even cross biological barriers (147). EVs are unique in protecting and delivering their internal cargo to target cells through ligand-receptor interactions. Previous studies have shown that proteins on EV surfaces facilitate cargo delivery and increase half-lives in circulation by promoting membrane fusion with the target cell and suppressing CD47-mediated phagocytic clearance, enhancing the pharmacological properties of EVs (148). In addition, EVs can load multiple substances, such as proteins, nucleic acids, and synthetic drugs, making them a versatile delivery platform. Thus, developing EVs as a drug delivery platform holds promise.

Cancer therapy represents one prominent application that requires a highly targeted drug delivery platform. Various chemotherapeutic agents can be loaded into EVs, including plant-derived natural compounds such as triptolide (TPL) and synthetic drugs such as paclitaxel. TNF-related apoptosis-inducing ligand (TRAIL) could selectively induce apoptosis of cancer cells, while the application of recombinant TRAIL protein faced the challenges of low bioavailability and resistance of cancer cells. However, EVs from TRAIL-expressing mesenchymal stem cells overcome these limitations, eliciting dose-dependent apoptosis in multiple cancer cell types *in vivo*, including pronounced apoptosis in TRAIL-resistant cancer cells (149). Another study utilizing TRAIL-expressing engineered EVs to encapsulate TPL demonstrated potent inhibition of tumor growth and reduced toxicity in a melanoma mouse model *in vitro* (150). MiRNAs also effectively modulate cancer progression; miR-195 is downregulated in drug-resistant melanomas. Tumor-derived EVs loaded with miR-195 reduced tumor volume *in vivo* and impaired engraftment and growth of xenografts implanted with melanoma cells exposed to MAPK inhibitors (151). Beyond the delivery of drugs and microRNAs to modulate cancer cells, cancer vaccines inducing tumor regression by triggering specific T-cell responses against tumor neoantigens represent another crucial aspect of cancer treatment. OMVs show promise as cancer vaccine candidates due to the enrichment of pathogen-associated molecular patterns, which can activate antigen-presenting cells to elicit effective antigen presentation and robust activation of the adaptive immune activation. Capitalizing on straightforward bacterial genetic editing and plug-and-display systems, OMVs can display diverse tumor antigens to customize cancer vaccines (152).

In addition to systemic administration, EVs can also be combined with biomaterials to achieve efficient localized administration for lesion sites. Hydrogels and scaffolds can be utilized to enhance the retention and therapeutic effects of EVs (153). For example, Self-assembling peptides (SAPs) are a class of biomaterials composed of natural amino acids that can spontaneously form nanoscale hydrogels under ionic saline conditions, exhibiting high biocompatibility (154). In a murine model of renal ischemia–reperfusion injury, mesenchymal stem cell-derived extracellular vesicles loaded into SAP hydrogel demonstrated greater therapeutic potential compared to a single dose of either EVs or SAP alone. This treatment improved renal function by reducing tubular cell apoptosis, pro-inflammatory cytokine expression, and macrophage infiltration (155).

## 6 Challenge and limitations of the application of EVs

The versatile applications of EVs in veterinary medicine are accompanied by inherent challenges that merit careful consideration. One major concern revolves around the lack of standardized procedures for EV isolation and preservation, given their inherent heterogeneity. This diversity poses difficulties in differentiating between vesicle subtypes and determining their origin, resulting in varying impurities and bioactive capabilities due to disparate isolation methods (33). The absence of a unified standard in isolation procedures introduces a notable level of inconsistency in research outcomes. This variability is particularly pronounced in diagnostic applications reliant on EV-based biomarkers, where challenges arise from potential similarities with circulating exosome miRNAs (51) and variations in protein and lipid content due to isolation methods (156). Preserving EVs, typically stored in PBS at −80°C, introduces challenges, with storage conditions impacting membrane structure efficiency. Achieving consensus on storage conditions and duration is pivotal for maintaining stable EV effects (157).

In addition, the challenge of using EVs for diagnosis tools can be difficult. Although there are business products available like *ExoDx Lung* by Exosome Diagnostics company reaching decent sensitivity and specificity, the reproducibility of EVs’ test results is still debatable, and it is difficult to compare test results across platforms horizontally, and the traceability chain of results is lacking (51). Especially for veterinary development, without the capability to recruit patients for large clinical trials, the task may be more challenging.

Further, the yield of functional EVs will also be problematic. A large part of EVs comes from cell culture medium, which limits the production. There are studies and methods to boost EVs production from cells, but still a gap in clinical translation (158). To enhance the therapeutic and targeting effect, the engineering process is needed. Various engineering methods were reported and the efficiency remains an obstacle. In veterinary medicine, where treatment doses are contingent upon animal size, the demand for large quantities further accentuates these challenges.

Lastly, cost control emerges as a significant hurdle in the application of EVs. The expense associated with obtaining EVs exceeds that of chemically synthesized medicines, and conserving bioactivity may necessitate additional transportation methods, posing a further economic consideration for treatment applications. Addressing these challenges necessitates concerted efforts in standardization, enhancing reproducibility, scalable production methods, and the formulation of cost-effective strategies to unlock the full potential of EVs in veterinary medicine.

## 7 Conclusion

EVs represent an emerging field of research in veterinary medicine, though investigations thus far remain largely preliminary. Current veterinary EV studies have principally focused on characterizing EVs across various physiological and pathological states, identifying potential vesicle-based biomarkers, and evaluating the therapeutic promise of EVs. As living standards and prosperity in the pet industry continue rising, demand for highly customized therapies creates a need for antibiotic-free alternatives and regenerative medicine solutions. Veterinary scientists would benefit from collaborating with human clinical medicine, where EVs have already been harnessed for clinical applications. Structured collaborative efforts between human and veterinary researchers will facilitate the translation of EVs into clinical veterinary practice to address emerging needs.

Overall, existing studies provide valuable foundational insights into the extensive potential of EVs as biomarkers and therapeutics in veterinary applications. However, continued investigations are needed to fully characterize EVs, elucidate mechanisms of action, and translate findings into clinical veterinary practice. Interdisciplinary cooperation and adherence to consistent protocols will be essential to properly evaluating, validating, and implementing EV-based veterinary diagnostics and therapies.

## Author contributions

YX: Writing – original draft. PL: Writing – review & editing. CX: Writing – review & editing. BH: Writing – review & editing. JL: Writing – review & editing. JG: Writing – review & editing.
